# Fibrin and poly(lactic-co-glycolic acid) hybrid scaffold promotes early chondrogenesis of articular chondrocytes: an *in vitro *study

**DOI:** 10.1186/1749-799X-3-17

**Published:** 2008-04-25

**Authors:** Munirah Sha'ban, Soon Hee Kim, Ruszymah BH Idrus, Gilson Khang

**Affiliations:** 1 Department of Physiology, Faculty of Medicine, Universiti Kebangsaan Malaysia, Jalan Raja Muda Abdul Aziz, 50300 Kuala Lumpur, Malaysia; 2 Tissue Engineering Laboratory, Universiti Kebangsaan Malaysia Hospital, 9th floor, Clinical Block, Jalan Yaacob Latif, 56000 Cheras, Kuala Lumpur, Malaysia; 3 BK-21 Polymer BIN Fusion Research Team, Department of Polymer Science and Technology, Chonbuk National University, 664-14, Dukjin, Jeonju, 561-756, Seoul, Korea

## Abstract

**Background:**

Synthetic- and naturally derived- biodegradable polymers have been widely used to construct scaffolds for cartilage tissue engineering. Poly(lactic-co-glycolic acid) (PLGA) are bioresorbable and biocompatible, rendering them as a promising tool for clinical application. To minimize cells lost during the seeding procedure, we used the natural polymer fibrin to immobilize cells and to provide homogenous cells distribution in PLGA scaffolds. We evaluated *in vitro *chondrogenesis of rabbit articular chondrocytes in PLGA scaffolds using fibrin as cell transplantation matrix.

**Methods:**

PLGA scaffolds were soaked in chondrocytes-fibrin suspension (1 × 10^6^cells/scaffold) and polymerized by dropping thrombin-calcium chloride (CaCl_2_) solution. PLGA-seeded chondrocytes was used as control. All constructs were cultured for a maximum of 21 days. Cell proliferation activity was measured at 1, 3, 7, 14 and 21 days *in vitro *using 3-(4,5-dimethylthiazole-2-yl)-2-, 5-diphenyltetrazolium-bromide (MTT) assay. Morphological observation, histology, immunohistochemistry (IHC), gene expression and sulphated-glycosaminoglycan (sGAG) analyses were performed at each time point of 1, 2 and 3 weeks to elucidate *in vitro *cartilage development and deposition of cartilage-specific extracellular matrix (ECM).

**Results:**

Cell proliferation activity was gradually increased from day-1 until day-14 and declined by day-21. A significant cartilaginous tissue formation was detected as early as 2-week in fibrin/PLGA hybrid construct as confirmed by the presence of cartilage-isolated cells and lacunae embedded within basophilic ECM. Cartilage formation was remarkably evidenced after 3 weeks. Presence of cartilage-specific proteoglycan and glycosaminoglycan (GAG) in fibrin/PLGA hybrid constructs were confirmed by positive Safranin O and Alcian Blue staining. Collagen type II exhibited intense immunopositivity at the pericellular matrix. Chondrogenic properties were further demonstrated by the expression of genes encoded for cartilage-specific markers, collagen type II and aggrecan core protein. Interestingly, suppression of cartilage dedifferentiation marker; collagen type I was observed after 2 and 3 weeks of *in vitro *culture. The sulphated-glycosaminoglycan (sGAG) production in fibrin/PLGA was significantly higher than in PLGA.

**Conclusion:**

Fibrin/PLGA promotes early *in vitro *chondrogenesis of rabbit articular chondrocytes. This study suggests that fibrin/PLGA may serve as a potential cell delivery vehicle and a structural basis for *in vitro *tissue-engineered articular cartilage.

## Background

Autologous chondrocytes implantation (ACI) was first published by Brittberg et al. [1] in 1994. This technique is quickly becoming a successful and viable alternative treatment in orthopaedic surgery to total knee replacement, arthroscopy, and abrasion therapy. Two-step procedures are required for ACI. After cartilage is biopsied and cultured, the next procedure is to implant cultured chondrocytes arthrotomically. The second procedure is invasive and have all of the risks associated with open surgery. Future improvements could be shifting the arthrotomy to arthroscopic procedure to help decrease the morbidity associated with arthrotomy. Therefore, we believed *in vitro *generation of 3D cartilage construct can be utilized to overcome the drawback. In recent years, several promising recovery of small full thickness lesions using *in vitro *3D cartilage constructs have been discovered in rabbit [2-4], goat [5,6], and dog [7]. We have successfully performed autologous 'chondrocytes-fibrin' construct (ACFC) implantation in sheep model [8-10] with good results. However during implantation, we still performed arthrotomy and used periosteum to hold the implant since ACFC was too soft to hold into defect independently. Therefore, basic research is still necessary to develop its full potential. Our next aim is to improve the scaffolding material of our *in vitro *3D cartilage construct.

Recently, various synthetic- and naturally-derived biodegradable polymers have been widely used to construct scaffolds for tissue engineering purposes [11,12]. Many trials have successfully cultured chondrocytes [13-15], reconstructed tissue engineered cartilage [16-19] and transplanted engineered cartilage into defect [3,8-10]. Thus, biocompatible scaffolds that afford cells proliferation and matrix accumulation have been widely investigated [2,20,21]. Advantages of synthetically-derived biodegradable polymers include controllable degradation rate, high reproducibility, and easy to fabricate into specific shapes. Whilst naturally-derived biodegradable polymers are usually mimicked the key elements of normal tissue [22].

Poly(lactic-co-glycolic acid) (PLGA) are bioresorbable and biocompatible synthetic polymer, rendering them as a promising tool for regenerative medicine and clinical application. Numerous attempts have been made for successful tissue reconstruction using PLGA-based scaffold either by PLGA itself [23,24] or in combination with natural polymers such as collagen [21,25], and extracellular matrices scaffolds, i.e. small intestinal submucosa [26,27] as well as demineralised bone particles [28]. Incorporation of bioactive molecules on PLGA surface is believed to mediate cells behavior, e.g. proliferation, differentiation and function [26-28]. To minimize cells lost during *in vitro *seeding procedure, we used fibrin to immobilize cells and to provide homogenous cells distribution in PLGA scaffolds. Until this article is written, apart from similar approach conducted by the research group from Germany [29-31], there is limited information with regard to the use of fibrin as a cell transplantation matrix for articular chondrocyte in PLGA. Previously, the use of fibrin gel immobilization technique resulted in homogeneous distribution and promoted bone formation of human periosteum-derived progenitor cells in PLGA [29], PLGA-TCP composites [30] and PLGA-polydioxanon fleeces [31]. Fibrin has also been used for cartilage reconstruction purposes [8-10,13-20]. We hypothesized that fibrin would be an ideal cell carrier/transplantation matrix and to enhance *in vitro *chondrogenesis of rabbit articular chondrocytes by mean of morphological, histological, biochemical and phenotypically similar to the normal hyaline cartilage.

## Methods

### Harvest of cartilage, chondrocytes isolation and monolayer culture expansion

Articular cartilage was aseptically dissected from the femoral condyles and patellae of 6 weeks-old New Zealand White rabbits (n = 6). Each sample was processed within 6 to 12 hours post-surgery. Cartilage was washed, minced and digested with 0.6% collagenase A (Roche Applied Science, Germany) at 37°C for 6 hours. Isolated chondrocytes were cultured at a density of 5,000 cells/cm^2 ^in F12 nutrient mixture (F12) and Dulbecco's Modified Eagle Medium (DMEM) (Gibco, Grand Island, NY) supplemented with 10% foetal bovine serum (FBS) (Gibco) with the presence of antibiotics and antimycotic (Gibco), 200 mM L-glutamine (Gibco) and 50 μg/ml of ascorbic acid (Sigma). All cultures were maintained in 5% CO_2 _incubator (Optima Model 560, Optima Inc, USA) at 37°C with the medium changed every other day.

### Preparation of microporous 3D PLGA scaffolds

PLGA copolymer (mole ratio 50:50, molecular weight 33,000 g/mole, Resomer RG 503 H) was purchased from Boehringer Ingelheim Pharma GmbH (Ingelheim, Germany). Micro-porous 3D PLGA scaffolds (0.2% w/v) were fabricated by the solvent casting/salt leaching technique using methylene chloride (CH_2_Cl_2_) (JT Baker, Baker Analyzed^® ^A.C.S reagent, Malaysia) as previously described [26,32]. Sieved sodium chloride (NaCl) particles (90 and 180 μm) were dispersed in a polymer/solvent solution, which was then cast to make a scaffold using cylindrical silicone moulds (7 mm in diameter and 3 mm thickness). The salt particles were then leached out by continuous soaking in deionized water for 48 hours. The scaffolds were freeze-dried for 48 hours using freeze-dryer (IlShin Lab Co. Ltd, South Korea).

### Formation of *in vitro *constructs

Each sample was assigned into two experimental groups – chondrocytes were seeded into (1) PLGA scaffolds with fibrin (fibrin/PLGA) and (2) PLGA without fibrin. Articular chondrocytes from primary passage (P0) were sub-cultured (P1) in 75 cm^2 ^culture flasks (Falcon). After confluence, cells were harvested, counted for total cell and viability. PLGA scaffolds were sterilized upon use by 70% ethanol. One million cells per scaffold was incorporated and resuspended with (1) fibrin glue kit from Greenplast^® ^(Green Cross P. D. Company, Yongin, Korea) and (2) culture medium. PLGA scaffolds were soaked in 'chondrocytes-fibrin' admixture and polymerized within 5 minutes by dropping thrombin-CaCl_2 _solution (Green Cross P. D. Company, Yongin, Korea). Chondrocytes suspension in culture medium was seeded directly into PLGA scaffolds. All constructs were cultured for 21 days *in vitro*. All constructs were evaluated at each time point of 1-, 2- and 3- weeks.

### Measurement of cell proliferation activity of *in vitro *constructs

Cell proliferation activity and cells viability was measured using MTT assay at day 1, 3, 7, 14 and 21 *in vitro*. The tetrazolium compound MTT (0.5 mg/ml) (thiazolyl blue tetrazolium bromide, Sigma-Aldrich Inc., St Louis USA) was added to all constructs and incubated for 4 hours at 37°C. The resulted crystals were solubilised by dimethylsulfoxide (DMSO) (Sigma Chemical Co., St Louis, USA). The absorbance was read using E-Max ELISA plate reader (Molecular Device, USA) at 570 nm – yielding absorbance as a function of viable cell number. Data was expressed as mean ± standard error of the mean (SEM). Results were analyzed using Student's t-test and the difference are considered significance when p < 0.05.

### Macroscopic observation, histology and immunohistochemistry analysis

Each construct was observed grossly at room temperature without any fixation and palpated with forceps to assess mechanical rigidity. After fixation with 10% formalin, specimens were processed and stained with Haematoxylin and Eosin (H&E) to assess tissue morphology, Safranin O to identify presence of proteoglycan-rich matrix and Alcian blue to detect accumulation of GAG. Immunohistochemistry (IHC) analysis was performed in accordance to the manufacturer's protocol (UltraTek HRP Kit, Immunotech, France) using monoclonal antibody (MAb) mouse anti-rabbit collagen type II (Calbiochem^® ^EMD Biosciences, Inc. La Jolla) and MAb mouse anti-rabbit collagen type I (Sigma Aldrich).

### Total RNA isolation, cDNA synthesis and conventional PCR

Total RNA was extracted from *in vitro *constructs at each time point of 1, 2 and 3 weeks using TRIzol reagent (Invitrogen, Carlsbad, CA) according to the manufacturer's protocol. Reverse transcription was carried out using Superscript™ II reverse transcriptase (Invitrogen, Carlsbad, CA) according to the manufacturer's protocol under the following conditions: 65°C for 5 minutes, 42°C for 2 minutes, 42°C and 70°C for 50 minutes and 15 minutes. Polymerase chain reaction was carried out using the Takara thermal cycler (Takara Bio Inc. Japan). Six-μl of the amplified PCR products were separated by 1.5% agarose gel electrophoresis (SeaKem^® ^LE Agarose, Cambrex Bio Science Rockland, Inc. USA), stained with SYBR^® ^green nucleic acid gel stain (Cambrex Bio Science Rockland, Inc. USA) and visualized by UV transillumination using gel documentation system EDAS 290 Kodak (Viber Lourmat, France). All primer sequences were as follows: collagen type II: forward: 5'-gcacccatggacattggaggg-3'/reverse: 5'-atgttttaaaaaatacgaag-3' [33]. Aggrecan core protein: forward: 5'-atcaacagagacctacgatgt-3'/reverse: 5'-gttagggtagaggtagaccgt-3' [34]. Collagen type I: forward: 5'-gatgcgttccagttcgagta-3'/reverse: 5'-ggtcttccggtggtcttgta-3' [33]. Rabbit β-actin gene [34] was used as an endogenous control: forward: 5'-ccggcttcgcgggcgacg-3'/reverse: 5'-tcccggccagccaggtcc-3'. All primers were prepared by GenoTech. Corp. (Daejeon, Korea).

### Sulphated glycosaminoglycan (sGAG) production assay

All samples were digested with papain digestion solution (125 μg/mL of papain, 5 mM L-cystein, 100 mM Na_2_HPO_4_, 5 mM EDTA, pH 6.8) at 60°C for 16 hours. Sulphated GAG contents were analyzed using a 1,9-dimethylmethylene blue (DMMB) assay [35]. Data was expressed as mean ± standard error of the mean (SEM). Results were analyzed using Student's t-test and the difference are considered significance when p < 0.05.

## Results

### Measurement of cell proliferation activity of *in vitro *constructs

**Figure 1:**
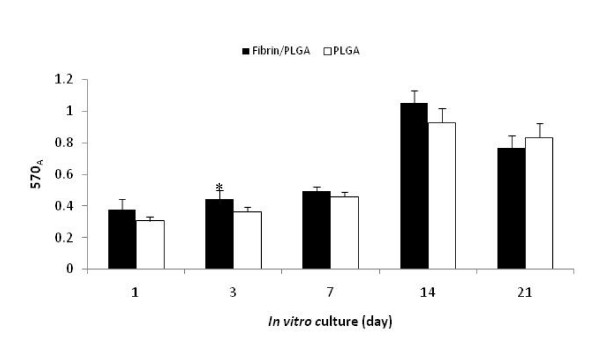
**Measurement of cell proliferation activity of *in vitro *constructs**. Fibrin/PLGA and PLGA construct exhibited similar growth pattern *in vitro*. Cells proliferation was gradually increased until day-14. Fibrin/PLGA showed a significant higher (*p *< 0.05) cells proliferation than PLGA at day-3 (*). Cells proliferation activity had declined by day-21.

Fibrin/PLGA hybrid construct and the PLGA group exhibited similar cell growth pattern *in vitro *(Figure 1). From the chart, cells proliferation was gradually increased from day-1 until day-7 with the fibrin/PLGA hybrid construct showed significantly higher cells proliferation activity (*p *< 0.05) compared to PLGA at day-3. Next, by day-14, cell proliferation activity in the fibrin/PLGA hybrid construct and PLGA constructs was significantly increased by 2.13-fold and 2.03-fold, respectively. However, the proliferation activity was then declined by day-21 in both groups. It has been indicated that the early stage of chondrogenesis involves the activity to establish cell-to-cell communication and cell-to-matrix interaction with regards to new cartilaginous tissue formation. We presumed at this stage the cellular proliferation has become less active. This could be one possible explanation in relation to the significant reduction of cell proliferation in fibrin/PLGA hybrid construct by 1.37-fold by the end of 21 days of *in vitro *culture.

### Macroscopic observation of *in vitro *constructs

**Figure 2:**
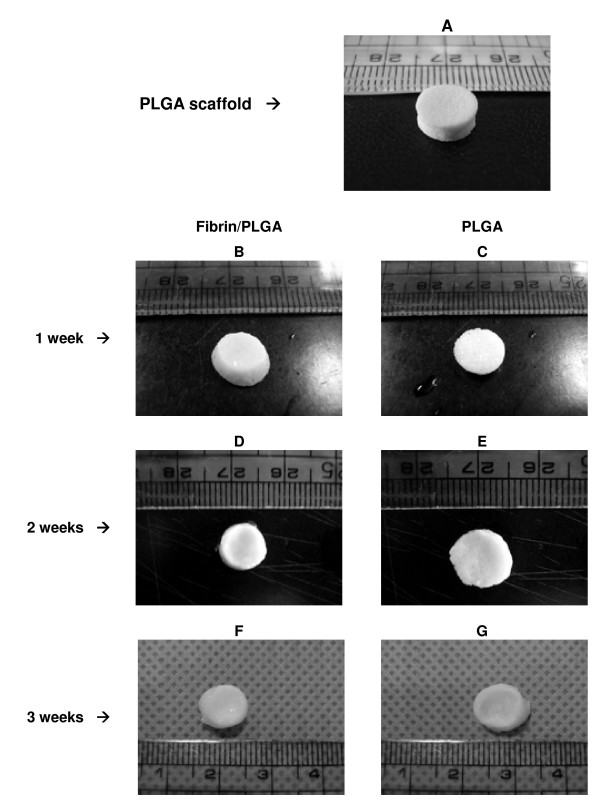
**Macroscopic observation of *in vitro *constructs**. Figure 2A represents PLGA scaffold which was designed in the shape of cylindrical disc. Fibrin/PLGA constructs (Figure 2B) and PLGA construct (Figure 2C) was morphologically similar after 7 days in culture. Fibrin/PLGA construct (Figure 2D) showed slightly smooth and glistening morphology when compared to PLGA (Figure 2E) after 14 days. By week 3, fibrin/PLGA construct appeared whiter, smoother and glistening (Figure 2F) than PLGA (Figure 2G).

PLGA scaffold was designed in the shape of cylindrical disc with 7 mm diameter × 3 mm height (Figure 2A). Scaffolds were prepared via solvent casting/salt leaching method. This selective dissolution technique produced highly porous polymer with pore sizes as same as the size of sieved NaCl granules (90 and 180 μm). Morphological appearance of *in vitro *fibrin/PLGA hybrid constructs (Figure 2B) and PLGA construct (Figure 2C) was similar by day 7 in culture. However, at day 14, fibrin/PLGA hybrid construct (Figure 2D) exhibited slightly smooth and glistening morphology when compared to PLGA construct (Figure 2E). Both constructs showed no resisting compression when palpated with forceps. By the end of the third week, the *in vitro *fibrin/PLGA hybrid construct appeared whiter, smoother and glistening (Figure 2F), resembling morphology of cartilage-like tissue superior to PLGA construct (Figure 2G). In addition, fibrin/PLGA hybrid construct was slightly firmer than the PLGA construct.

### Histological evaluation of *in vitro *constructs

**Figure 3:**
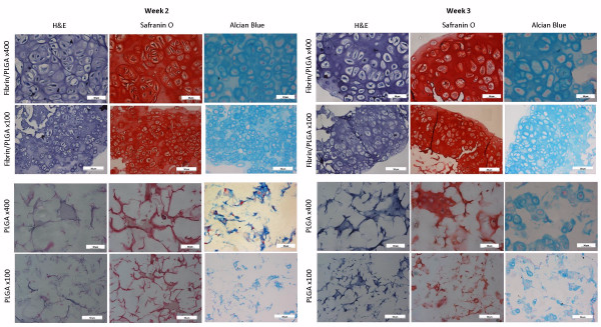
**Histological evaluation of *in vitro *constructs**. Fibrin/PLGA constructs showed superior histological features of cartilage-like tissue compared to PLGA. Differences between fibrin/PLGA (Figure 3A, B, C and Figure 3G, H, I) and PLGA (Figure 3D, E, F and Figure 3J, K, L) were clearly visible in term of overall cartilaginous tissue formation, cells organization and ECM distribution. The fibrin/PLGA constructs was intensely stained with Safranin O for accumulated proteoglycan and Alcian Blue for GAG at 2 weeks and greatest at 3 weeks.

At 2 weeks *in vitro*, when the fibrin/PLGA hybrid construct were stained using H&E, they predominantly showed superior histological features of normal cartilage compared to the PLGA group. The closely-packed cartilage-isolated cells were homogeneously distributed in the ECM and exhibited rounded morphology with lacunae embedded in basophilic ground substance (Figure 3A). The pericellular and inter-territorial matrix region was strongly stained by the characteristic red of Safranin O, indicating presence of the proteoglycan-rich matrix (Figure 3B) corroborated with positive Alcian Blue staining (Figure 3C) confirming GAG accumulation. Next, the formation of cartilaginous tissue was remarkably evident by the third week of *in vitro *culture in the fibrin/PLGA hybrid construct. Cartilage-isolated cells with lacunae was well-distributed within the homogenous ECM (Figure 3G) in concert with the presence of specific histochemicals property of proteoglycan-rich matrix (Figure 3H) and GAG accumulation (Figure 3I). The difference between the fibrin/PLGA hybrid construct (Figure 3A, B, C and Figure 3G, H, I) and PLGA group (Figure 3D, E, F and Figure 3J, K, L) was clearly visible in term of overall cartilaginous tissue formation, cells organization and ECM distribution in all specimens. PLGA group exhibited few rounded chondrocytes cluster filling up several void spaces of the scaffold. For fibrin/PLGA hybrid construct, accumulation of proteoglycan-rich matrix and GAG at the core region was significant and was intensely stained at 2 weeks and greatest at 3 weeks when compared to PLGA construct. No sign of cartilaginous tissue formation in fibrin/PLGA hybrid construct and PLGA construct was observed at one week of *in vitro *culture.

### Immunohistochemistry analysis of *in vitro *constructs

**Figure 4:**
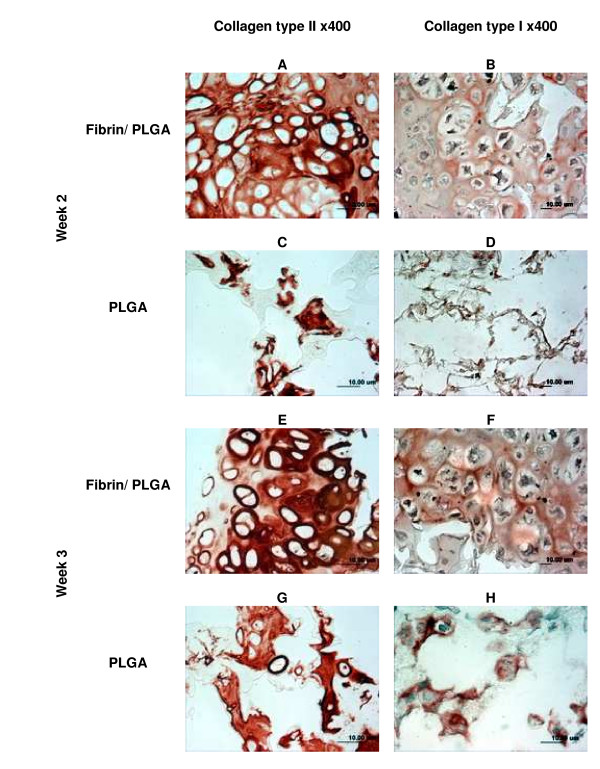
**Immunohistochemistry analysis of *in vitro *constructs**. As shown in Figure 4A, fibrin/PLGA exhibited strong immunopositivity of collagen type II which mainly localized at the pericellular and inter-territorial matrix. Minimal collagen type II expression could be observed in the PLGA construct (Figure 4C). After 3 weeks, collagen type II expression was maintained in fibrin/PLGA (Figure 4E) and PLGA (Figure G). Collagen type I in fibrin/PLGA constructs showed moderate immunopositivity at week-2 (Figure 4B) and week-3 (Figure 4F), as did PLGA (Figure 4D, Figure 4H).

We analyzed collagen type II and collagen type I immunolocalization on the fibrin/PLGA hybrid construct, and we compared the results with the PLGA group. The specific cartilaginous ECM molecule, collagen type II exhibited strong immunopositivity at the pericellular and the inter-territorial matrix of the fibrin/PLGA hybrid constructs (Figure 4A). Minimal collagen type II expression was observed in PLGA specimens (Figure 4C). After 3 weeks, as shown in Figure 4E collagen type II marker maintained positive expression in the fibrin/PLGA hybrid construct, as did the chondrocytes cluster in PLGA construct (Figure 4G). Collagen type I expression demonstrated moderate immunopositivity throughout the ECM of both fibrin/PLGA hybrid constructs (Figure 4B, Figure 4F) and the PLGA group (Figure 4D, Figure 4H) at week 2 and week 3, respectively.

### Cartilage-specific phenotypic expression analysis

**Figure 5:**
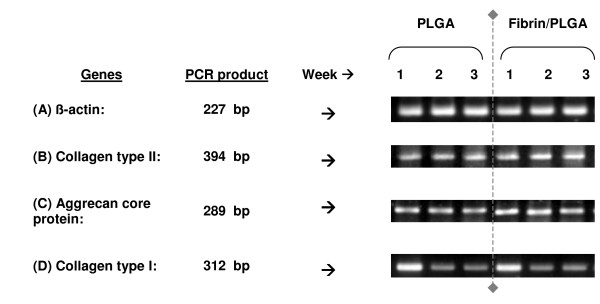
**Cartilage-specific phenotypic expression analysis**. The expression of genes encoded the cartilage-specific markers; collagen type II and aggrecan core protein was steadily expressed in fibrin/PLGA and PLGA. Interestingly, suppression of collagen type I was observed in fibrin/PLGA and PLGA at 2 weeks and 3 weeks. β-actin gene was steadily expressed in all samples to verify the analysis was reliable and successful.

When the mRNA expression of fibrin/PLGA hybrid construct and PLGA group were compared, no significant difference was observed between chondrocytes derived from both groups. The fibrin/PLGA hybrid construct and PLGA group showed comparable potential in sustaining the specific chondrogenic phenotypic expression at each time point of 1, 2 and 3 weeks. The expression of genes encoded the cartilage-specific markers; collagen type II and aggrecan core protein was steadily observed in *in vitro *culture, whereas collagen type I, the cartilage dedifferentiation marker exhibited down-regulation pattern after 2 and 3 weeks *in vitro*. The house-keeping gene, β-actin was steadily expressed in all specimens; to verify the two-step reverse-transcriptase PCR analysis was reliable and successful. Results were summarized in Figure 5.

### Sulphated glycosaminoglycan (sGAG) production assay

**Figure 6:**
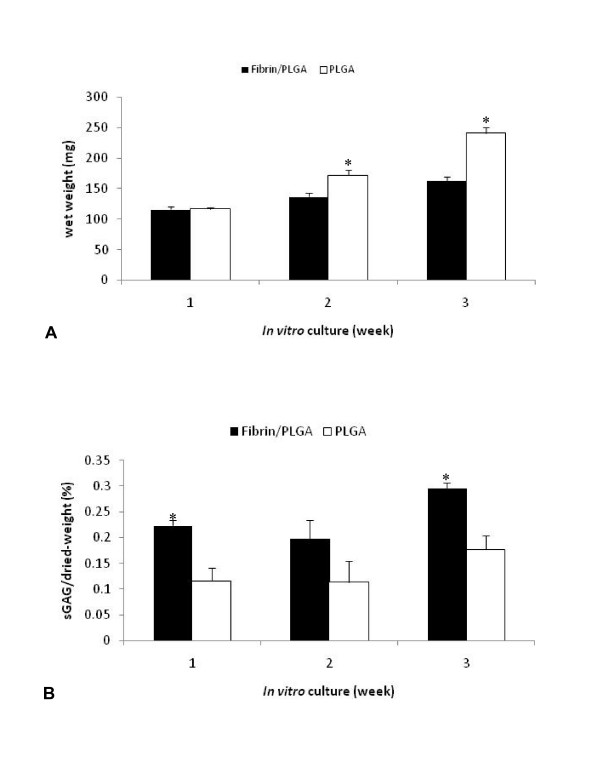
**Sulphated-glycosaminoglycan (sGAG) production assay**. The wet weight (Figure 6A) and sGAG production (Figure 6B) of the *in vitro *constructs were measured at 1, 2, and 3 weeks of culture, respectively. After 2 and 3 weeks *in vitro*, PLGA demonstrated significantly higher wet weight (*p *< 0.05) compared to fibrin/PLGA. The sGAG production in fibrin/PLGA construct was superior to PLGA. Relative sGAG contents (%) were significantly higher (*p *< 0.05) in fibrin/PLGA than PLGA at 1 week and 3 weeks.

The increment of average wet weight of fibrin/PLGA hybrid constructs (116.27 ± 4.65 mg, 137.25 ± 6.08 mg, 162.69 ± 7.12 mg) and PLGA group (116.88 ± 1.98 mg, 172.20 ± 8.78 mg, 241.33 ± 9.82 mg) was statistically significant (*p *< 0.05) from week 1, week 2 and week 3, respectively. After 2 and 3 weeks of *in vitro *culture, the PLGA group demonstrated significantly higher wet weight (*p *< 0.05) than fibrin/PLGA hybrid constructs by 1.25-fold and 1.48-fold, respectively (Figure 6A). As shown in Figure 6B, sGAG production in the fibrin/PLGA hybrid construct was definitely superior to the PLGA group at each time point. Normalized by the dried-weight of each sample, the relative sGAG content (%) was significantly higher (*p *< 0.05) in fibrin/PLGA hybrid constructs compared to the PLGA group at 1 week and 3 week cultures. In particular, at week 1, with 0.223 ± 0.010 relative sGAG content, fibrin/PLGA hybrid constructs exhibited 1.92-fold higher sGAG production than the PLGA group; 0.116 ± 0.025. At week 2, the relative sGAG content in fibrin/PLGA hybrid constructs; 0.197 ± 0.037 seemed higher than 0.113 ± 0.042, the relative sGAG content in PLGA group; however the magnitude showed no significance difference between both groups. Next, by week 3, fibrin/PLGA hybrid constructs exhibited 0.296 ± 0.011 relative sGAG content, which was 1.67-fold higher than 0.177 ± 0.027 relative sGAG content in the PLGA group.

## Discussion

Our aimed was to evaluate *in vitro *chondrogenesis of rabbit articular chondrocytes in PLGA scaffold utilizing fibrin as a cell transplantation matrix. Fibrin is biodegradable, biocompatible and non-immunogenic natural material [36], thus rendering this material as suitable scaffolding cell carriers [20] that helps provide homogenous cells distribution with no significant cells lost during the seeding process [29-31]. Immobilization of chondrocytes in fibrin resulted in homogenous cells distribution in PLGA scaffolds, easy to handle and deliver the cells [37]. Similar finding was reported in the previous assessment of osteogenic potential utilizing human periosteum-derived progenitor cells and fibrin gel immobilization technique in PLGA scaffold [29-31]. With regards to the present study, Lee et al. [37] also reported fibrin provided more uniform chondrocytes distribution during cell seeding via histology in macro-porous polyurethane scaffold.

Recently, Endres et al. [38] showed the 3D arrangement of human articular chondrocytes in human fibrin glue and resorbable PGA scaffolds cultured in the presence of human serum is an excellent system for the maturation of cartilage grafts in articular cartilage regeneration. It has been well documented that during growth in monolayer culture, chondrocytes adopt many of the phenotypic traits of fibroblast, as they become elongated and synthesize type I collagen rather than type II collagen. Thus, to induce the re-differentiation of expanded chondrocytes, the cells were first combined with fibrin glue as a temporary matrix and embedded in a resorbable felt structure to achieve a three-dimensional environment [38]. In this study, following cells seeding onto scaffolds, cells proliferated markedly in fibrin/PLGA and PLGA. Because of the growth, chondrocytes can secrete appropriate ECM molecules and develop chondrocyte-chondrocyte interaction to form clusters of various sizes as well as the 3D structure while preserving the original shape of the cell. By 2 weeks of culture period, histological differences between fibrin/PLGA and PLGA were obviously developed. Newly formed ECM was concentrated around the rounded cells, consistent with the established notion that a rounded morphology is an obligatory for the chondrocytic phenotype. Besides the histologically mature chondrocyte, extensive development of ECM indicated by presences of abundant proteoglycan-rich matrix and accumulated GAG in fibrin/PLGA was better than in PLGA. The expression of collagen type II, cartilage-specific ECM molecule was noticeably superior in fibrin/PLGA compared to PLGA. By day 21, fibrin/PLGA had significant cells-matrix organization and ECM deposition compared to PLGA group. Decline in growth rate by 21 days can be explained by a morphologically and structurally stable cells-matrix organization entering a steady state with no active cellular function at this stage. Clearly, the ECM production on fibrin/PLGA was superior to that of PLGA group. Lee et al. [37] suggested that the phenomenon may be due to higher cell-seeding efficiency and more homogeneous distribution of chondrocytes in the fibrin/PLGA hybrid construct. Similar criterion could be observed in PLGA-incorporated with collagen micro-sponges which was previously encountered as a promising 3D scaffold for articular cartilage tissue engineering [21,25].

Although there were remarkable histological differences in fibrin/PLGA hybrid scaffold and PLGA group, there was no significant variation in the semi-quantitative gene expression assessment for collagen type II, aggrecan core protein and collagen type I. Gene expression profiles showed that the chondrocyte phenotype was maintained in both groups. Interestingly, suppression of cartilage dedifferentiation marker, collagen type I can be observed in the *in vitro *constructs. Previously, although Lee et al. [37] reported the fibrin hydrogel-polyurethane hybrid scaffold system promoted higher levels of cartilage gene expression in the early stage of culture, the system still did not permit maintenance of the chondrocyte phenotype for the entire 4-week culture period. Accordingly, we suggest that fibrin would be an ideal cell carrier/transplantation matrix and enhance *in vitro *chondrogenesis of rabbit articular chondrocytes by mean of morphological, histological, biochemical and phenotypically similar to the normal hyaline cartilage. If this result is applicable for the clinical use, it is practically reliable for the reconstruction of clinical transplants for future orthopaedic surgery.

## Conclusion

Fibrin/PLGA hybrid scaffold promotes early *in vitro *chondrogenesis of rabbit articular chondrocytes proven by means of morphology, histology, immunohistochemistry, chondrogenic gene expression and sGAG production. This study suggests that fibrin/PLGA hybrid scaffold may serve as a potential cell delivery vehicle and a structural basis for *in vitro *tissue-engineered articular cartilage construct. The *in vivo *experiment has been carried out and the results are currently written as a next chapter for this study.

## Competing interests

The authors declare that they have no competing interests.

## Authors' contributions

**MS **conceived the study, participated in its design, performed all the experiments and drafted the manuscript. **SHK **participated in the design of the study and conceived of the study. **RBHI **participated in the design of the study and conceived of the study. **GK **participated in the design of the study, conceived the study and drafted the manuscript. All authors read and approved the final manuscript.
